# Book Reviews

**Published:** 1888-04

**Authors:** 


### BOOK-REVIEWS.

THE RULES OF ASEPTIC AND ANTISEPTIC SURGERY. A Practical Treatise for the Use of Students and the General Practitioner. By ARPAD G. GERSTER, M. D. Pp. vi and 332. New York: D. Appleton & Co. 1888. Chicago: A. C. McClurg & Co.

"Prevention has become the watchword of modern practice, and it can be said that, by the successful employment of the preventive methods of the present day, surgery has become a conservative branch of the healing art." This treatise is the result of an attempt on the part of the author to illustrate the truth of the quotation given, and of the following one, both from the preface. "It can not now be successfully denied that the surgeon's acts determine the fate of a fresh wound, and that its infection and suppuration are due to his technical faults of omission and commission." The truth of this last statement is just as unhesitatingly admitted by all qualified surgeons to-day as it would have been unhesitatingly denied by equally qualified men twenty years ago. Indeed this fact in regard to suppuration in fresh wounds is so fundamental that no one who doubts its truth ought to practice plastic surgery. The fact that many practitioners do their surgical work either in ignorance or disregard of this principle is ample excuse for the stress laid upon it in this connection.

The book is subdivided into five parts: Asepsis, Antisepsis, Tuberculosis, Gonorrhœa, and Syphilis. As this division of the general subject indicates, the discussion of the special subjects of surgery is both new and unique. The author particularly disclaims any attempt at completeness—"the inclusion of al of the disciplines of surgery"—but to illustrate the changes in wound treatment in practical surgery, and "to place before the profession a vivid and true picture of contemporaneous surgical methods." As far as possible the principles discussed are illustrated by actual cases taken from the author's practice.

The illustrations, a special feature of the book, are from photographs of actual cases, and demonstrate the steps of the operations described, from the application of the aseptic protective bandages and dressings, applied before beginning, to the completed dressing at the close of the operation, when the patient is ready for removal. No illustrations could be more striking of the difference between the old and the new practice. They are prepared by the photo-engraving process and resemble actual photographs very strongly. This method of illustration of medical books is new, and is a welcome innovation. There are two hundred and forty-eight of the illustrations, and, with few exceptions, they are not pictures of instruments.

The author is to be congratulated, both upon the undertaking and upon the success with which he has accomplished it. Instead of containing a lot of material which all others upon the same subject contain, it is filled with matter which no other book of its kind contains. It is not exaggerated praise to say that it is the most important contribution to surgical literature which has appeared in this country in recent years.

The manner in which the publishers have done their work is beyond unfavorable criticism.

E. W.

THE PRACTICE OF MEDICINE AND SURGERY APPLIED TO THE DISEASES AND ACCIDENTS INCIDENT TO WOMEN. By W. H. BYFORD, A. M., M. D., Professor of Gynæcology in Rush Medical College, and of Obstetrics in the Woman's Medical College, etc., and HENRY T. BYFORD, M. D., Surgeon to the Women's Hospital, of Chicago; Gynæcologist to St. Luke's Hospital; President of the Chicago Gynæcological Society; Member of the American Medical Association, of the Illinois State Medical Society, and of the Chicago Medical Society, etc. Fourth edition; revised; re-written and very much enlarged; with 306 illustrations. Philadelphia: P. Blakiston, Son & Co. 1888.

It is hardly necessary to say anything of this work, except that the additions and revisions bring it fully up to the date of publication. Dr. Byford is so well known, and his work has been so generally accepted as one of the very best upon the subjects of which it treats, that the simple announcement of a new edition might suffice. We can not, however, omit the opportunity to note the views of the authors upon the subject of oöphorectomy. As to the results of the operation, or rather the nature of the change accomplished, it, of course, arrests ovalation, and, in a large majority of cases, uterine hæmorrhage or menstruation. This produces menopause, but, say the authors, "we should not forget that menopause is not change of life."

"Senile menopause, one of the symptoms of the change of life, is the consequence of gradual changes in all the organs concerned. This change is a degeneration of the genital organs." The question of the influence of the ovaries upon the nutrition of other portions of the sexual apparatus is touched upon, especially in its bearings upon uterine fibroids, as follows: "The removal of the ovaries in the presence of a large fibroid and hypertrophied uterus, simply takes away their governing agency before the process of degeneration has begun." A doubt is expressed as to the value of such a procedure in the case supposed. The conclusion of a discussion of the difference between the effects of a natural change of life and oöphorectomy upon fibrous tumors of the uterus is in the following words: "I do not wish to be understood as opposing oöphorectomy; they (the reflections), however, make me hesitate to give an unconditional adhesion to the practice, even when in our present knowledge it would seem indicated."

"For intolerable and incurable cases of oöphoroneurosis" the authors, however, consider the operation as often most helpful. Even here a careful inquiry should be made as to the cause or causes of the condition, with a view to remedy the trouble by some other means. They say: "Then the question comes up, whether we ought to spay our patient or prescribe and enforce the proper amount and kind of primitive living necessary to revolutionize her nervous functions?" It will gratify a large number of the members of our profession to see upon the title page the name of Dr. Henry T. Byford. The careful reader, acquainted with his contributions to the literature of this specialty, will recognize his valuable assistance in the work of revision and rewriting.

RECTAL AND ANAL SURGERY; WITH A DESCRIPTION OF THE SECRET METHODS OF THE ITINERANTS. By EDMUND ANDREWS, M. D., LL. D., Professor of Clinical Surgery in the Chicago Medical College, Senior Surgeon to Mercy Hospital, and E. WYLLYS ANDREWS, A. M., M. D., Adjunct-Professor of Clinical Surgery in the Chicago Medical College, Surgeon to Mercy Hospital. With original illustrations. Chicago: W. T. Keener. 1888.

This little book, of 111 pages, has been written and published to answer two questions concerning the diseases of the anus and rectum: "1. What are the best methods of diagnosis and treatment of these affections known to the regular profession? 2. What are the secret methods of the itinerants, and what is their value?"

The work does not pretend to be an exhaustive treatise upon the etiology, pathology, etc., of these troubles, but it is a well-condensed statement of the established opinions and methods of the best authorities upon a subject which has been greatly neglected by the profession, and which has, therefore, in the language of the authors "given charlatans an opportunity to slip in and occupy it in pestiferous numbers." The exposure of the secret methods of the traveling "pile doctor" is an amusing bit of history.

Nasal Polypus, with Neuralgia, Hay-Fever and Asthma, in Relation to Ethmoiditis. By Edward Woakes, M.D., London, Senior Aural Surgeon and Lecturer on Diseases of the Ear at the London Hospital; Surgeon to the London Throat Hospital. With illustrations. 8vo. Pp. 140. Philadelphia: P. Blakiston, Son & Co. Chicago; W. T. Keener. 1887.

This monograph upon ethmoiditis and its consequences, is based upon the extensive personal observations of the author, and bears evidence of careful study of the different stages and various forms of this affection, together with its anatomical and physiological consequences, The author's views are well matured and his deductions are clear. His contributions to our knowledge of the pathological conditions leading to polypoid growths are of special interest.

Lessons in Gynæcology. By William Goodell, A. M., M. D., Professor of Clinical Gynæcology in the University of Pennsylvania, etc. Third edition; thoroughly revised and greatly enlarged; with 112 illustrations. Philadelphia: D. G. Brinton.

The second edition of Dr. Goodell's valuable work has been for some time out of print, and the profession will be glad to know that the author has found time to revise and present to the student the latest views of the leading authorities in this specialty, but more particularly will the busy practitioner be glad to have the matured judgment of Dr. Goodell himself upon the vexed problems that are just now claiming the attention of the profession.

Of oöphorectomy, or "Battey's operation," he says: "In well-selected cases, this operation has been followed by wonderful results, but," he adds, "it has been greatly abused." He cites cases illustrating his views as to the indications for the operation, and discusses at some length the results of the removal of the ovaries upon the physical, psychical, and social conditions of the patient. Upon the whole, he concludes that the effect is about the same as that of the normal menopause.

The conclusion of the article is as follows:

"The operation of spaying is yet in its infancy, and time is needed to develop its resources. From being performed too frequently and without sound warrant, it is in danger of falling into disrepute. Yet, I can not but feel that in carefully selected cases it will prove the sole means of curing many mental and physical disorders of menstrual life, which have hitherto baffled our science, and are a standing opprobrium to our profession."

The final lesson is devoted to a consideration of the causes of uterine disorders.

Diseases of the Heart and Circulation in Infancy and Adolescence. By John M. Keating, M. D., Obstetrician to the Philadelphia Hospital, etc., etc., and William A. Edwards, M. D., Instructor in Clinical Medicine and Physician to the Medical Dispensary in the University of Pennsylvania, etc., etc. Illustrated with photographs and wood engravings. Philadelphia: P. Blakiston, Son & Co. 1888. Chicago: W. T. Keener.

This work consists of articles published by the authors in the Archives of Pediatrics during the year 1887. In collecting and publishing them in book form they have rendered a valuable service to the profession. Works on diseases of the heart in general are often unsatisfactory in their treatment of the derangements that so often occur during the growing and developing period of life. The information and data here collected will prove especially useful to the general practitioner. The book is not large—212 pages—and yet contains the sum of what is known upon the subject of which it treats.

A Synopsis of the Physiological Action of Medicines. Prepared for the use of the Students of the Medical Department of the University of Pennsylvania, with the approval of the Professor of Materia Medica. By Louis Starr, M. D., Clinical Professor of Diseases of Children in the Hospital of the University of Pennsylvania, and James B. Walker, M. D., Professor of Practice of Medicine in the Woman's Medical College, Philadelphia, assisted by W. M. Powell, M. D., Professor to the Clinic for Diseases of Children in the Hospital of the University of Pennsylvania. Third edition, enlarged. Philadelphia: P. Blakiston, Son & Co. 1888.

The book contains outlines of the physiological action of thirty-four drugs, the most of them being in common use. The arrangement of the work is systematic. Its scope is limited, and the matter is given in a very condensed form.

The fact that the book has reached a third edition indicates that there are many who are appreciative of such favors.

Chemical Analysis of Healthy and Diseased Urine, Qualitative and Quantitative. By T. C. Van Nüys, Professor of Chemistry, Indiana University. With thirty-nine wood-engravings. 8vo. Pp. 187. Philadelphia: P. Blakiston, Son & Co. Chicago: W. T. Keener.

This excellent book is quite a complete manual of chemical urinalysis. It is well adapted to the laboratory—better than to the needs of the busy physician—since it is confined almost entirely to the more precise methods, especially in the part devoted to quantitative estimations, omitting all mention of other simpler, more convenient, and approximately accurate tests in extensive use.

